# Enlarged Prostate Mistaken for Calculus

**Published:** 1842-10-01

**Authors:** 


*Enlarged Prostate mistaken for Calculus.*—M. Ripault, of Dijon, was called to assist at an operation for stone. The patient was an adult, in whom all the usual symptoms of calculus were present: the characteristic sound occasioned by striking the sound against a hard body was distinctly heard by several persons, and the existence of a stone undoubted. The lateral operation was performed, the bladder was opened; but the forceps, several times introduced, brought away nothing but clots of blood. An encysted stone was suspected, but the operation was discontinued. Nevertheless, the pains, which the patient previously experienced, ceased for some months; after which they recurred with greater violence; difficulty of micturition increased, the urine became ammoniacal, fever set in, and the patient died six months after the operation. On examination of the body, the pustules were found enlarged and composed of tough fibrous tissue, a section of which had a horny appearance, and when struck with the sound it communicated the same sensation which had been experienced during life.—*Ibid, from the Académie des Sciences.*

